# High Adherence to the Mediterranean Diet Is Associated with a Reduced Risk of Obesity among Adults in Gulf Countries

**DOI:** 10.3390/nu13030995

**Published:** 2021-03-19

**Authors:** Israa M. Shatwan, Eiman A. Alhinai, Balqees Alawadhi, Shelini Surendran, Najlaa M. Aljefree, Noha M. Almoraie

**Affiliations:** 1 Food and Nutrition Department, Human Sciences and Design Faculty, King Abdulaziz University, Jeddah 3270, Saudi Arabia; naljefree@kau.edu.sa (N.M.A.); nalmorie@kau.edu.sa (N.M.A.); 2 Dietetics Department, Al Nahdha Hospital, Ministry of Health, Ruwi 937, Muscat PC 112, Oman; iman.alhinai@moh.gov.com; 3 The Public Authority of Applied Education and Training (PAAET), Department of Food and Nutritional Sciences, College of Health Sciences, Shuwaikh, Kuwait; b.alawadhi@paaet.edu.kw; 4 Faculty of Health and Medical Sciences, University of Surrey, Guildford, Surrey GU2 7XH, UK; s.surendran@surrey.ac.uk

**Keywords:** Mediterranean diet, score, obesity, body mass index, gulf countries, Saudi Arabia, Kuwait, Oman

## Abstract

The Mediterranean diet (MedDiet) is considered as a good example of a healthy dietary pattern that has protective effects on obesity. The aim of the present study was to assess the adherence of adults from three Gulf countries (Saudi Arabia, Oman, and Kuwait) to the MedDiet and its association with obesity risk. A cross-sectional study was conducted on 961 men and women (75.7%) aged 20–55 years old. Waist circumference (WC), and hip circumference (HC) were measured waist/hip ratio (WHR) and body mass index (BMI) were calculated. A validated 14-item Questionnaire was used to measure adherence to MedDiet. The mean of the adherence to MedDiet score was 5.9 ± 2.03 for the total sample. An inverse association was observed between the adherence to MedDiet and BMI after adjusting for potential confounders (*p* = 0.0003 in total participants, and *p* = 0.001 in women only). A protective effect was seen with a higher adherence to the MedDiet on HC, suggesting that a greater adherence to the MedDiet was associated with a decreased HC (*p* = 0.04 in total participants, and *p* = 0.01 in women only). In conclusion, low adherence to the MedDiet among participants from three gulf countries was associated with increased obesity indicators, BMI, and HC.

## 1. Introduction

Obesity is a global public health crisis that has reached epidemic proportions [1]. The rising trends in body mass index (BMI) has accelerated the morbidity and mortality of chronic diseases in Asian populations [2,3], among them, the Gulf region population has had the most dramatic increase. The percentage of overweight and obese individuals among Gulf countries population is 60% and 30%, respectively [4]. Furthermore, in the Gulf region, Kuwait is the highest ranked country in terms of obesity, followed by Saudi Arabia, while Oman is reported to have the lowest rates of obesity in the region [5]. BMI is a well-known indicator for obesity status; however, it may lead to inconsistent results, since it does not discriminate between fat and muscle mass. There are many other obesity indicators such as waist circumference (WC), hip circumference (HC), and waist/hip ratio (WHR), which are considered as a better predictor of abdominal obesity, which consequently corresponds with a high incidence of cardiovascular disease [4]. Studies to date suggest that, compared with White Caucasians, abdominal and visceral adiposity is greater among Asian populations of the same body weight. [3]. The rapid increase in obesity can be explained by environmental factors, including physical inactivity and dietary patterns [6].

Dietary patterns among Saudi individuals have been investigated in different regions, age groups, and both sexes. Findings have demonstrated a high consumption of fast food and carbonated drinks, especially among the younger population. A low consumption of dairy products [7,8,9,10] and whole grains [11] was observed, with inconsistent results regarding fruit and vegetable consumption [7,8,10]. Data from the Saudi Health Interview Survey (*n* = 10,735) showed that 5.2% of individuals met dietary guideline recommendations for fruits, 7.5% for vegetables, 31.4% for nuts, and 44.7% for fish consumption. Moreover, the consumption of processed foods and sugar-sweetened beverages was high in Saudi adults [12]. In Kuwait, dietary patterns were characterized by high intake of rice, vegetables, fast food, and refined grains, lamb, and poultry but low of fruit, especially among younger adults compared to older adults [13,14]. In Oman, consumption of dairy products and vegetables were inadequate, while consumption of cereals, fruit, fish, meat, and chicken were adequate in elderly individuals [15]. A study on Omani adolescents showed low fruit and vegetable intake and high intake of sugar-sweetened beverages [16]. These consumption patterns are associated with an increased risk of obesity among Gulf countries inhabitants [8,17,18,19].

Recently, there has been a shift in research interests from testing the effects of individual nutrients and food components, into considering the overall dietary patterns on health status [20]. In order to study the adherence to such a dietary pattern, two methods can be utilized. First, cluster and factor analysis and second, indices and scores such as Mediterranean Diet Score [21,22]. The Mediterranean diet (MedDiet) is mainly composed of vegetables, fruits, legumes, fish and seafood, nuts and cereals, and unsaturated fatty acids (found in olive oil). However, meat and its constituents are not used often. The MedDiet is considered high in its content of dietary fibers, antioxidants, and minerals such as β-carotene, vitamin C, tocopherols, and omega 3 fatty acids [23], as well as, high amount of monounsaturated fatty acids and polyunsaturated fatty acids, while low of saturated fatty acids and trans fatty acids contents. Hence, it has been confirmed that the MedDiet can be considered as a good example of a healthy dietary pattern [24], due to its preventative effects of non-communicable diseases such as cardiovascular diseases, obesity, type 2 diabetes, and cancer [25]. Findings from randomized control trials demonstrated that participants who were assigned to a MedDiet had a reduction in BMI (−0.29 kg/m^2^) and body weight (−0.29 kg), compared to participants who were assigned to control diets [26]. Additionally, several European cohorts have showed that individuals with a higher adherence to MedDiet are associated with lower weight gain, compared to individuals with a lower adherence [27,28].

Previously, researchers related to the MedDiet, have only focused on the Mediterranean regions [27,29,30,31] and less on other geographically distant areas that are socially, environmentally, and economically different [32]. To our knowledge, limited studies have assessed adherence to the MedDiet in Saudi Arabia [33], but no previous study has compared the adherence to the MedDiet among Gulf countries and have examined the association between adherence to the MedDiet and obesity among Gulf populations. Therefore, the aim of the present study was to measure adherence to the MedDiet among adults from three Gulf countries (Saudi Arabia, Oman, and Kuwait) using a 14-validated item questionnaire and to examine its association with obesity risk using several obesity indicators body mass index (BMI), waist circumference (WC), hip circumference (HC) and waist to hip ratio (WHR).

## 2. Materials and Methods

### 2.1. Study Population

The study is a cross-sectional study. Participants were recruited from three Gulf countries Saudi Arabia, Oman, and Kuwait, during 2020. A total of 961 men and women aged 20–55 years old were engaged in the current study. Saudi participants were recruited from King Abdulaziz University, Jeddah, Saudi Arabia, Omani from Masqat city, and Kuwaiti from Kuwait City. The exclusion criteria included the presence of any metabolic diseases (such as diabetes, cardiovascular diseases, hypertension, liver disease, renal failure, cancer, or gastrointestinal disorder) based on self-report, having any type of medical therapy, and women who are pregnant or lactating. This study was approved by Unite of Biomedical Ethics Research Committee at King Abdulaziz University (Reference No 288–20) for the Saudi data, Kuwait Food and Nutrition Association for the Kuwait data, and Research and Ethical Review & Approval Committee, Ministry of Health for the Omani data (MOH/CSR/20/23684).

### 2.2. Data Collection

The study used a self-administered survey which consisted of three parts. The survey was distributed online through a web link shared over students’ university emails and WhatsApp groups, this approach was used due to closure of universities during the COVID-19 pandemic, for both Saudi and Kuwaiti participants. In Oman, a web link of the survey was shared over Omani nutrition and dietetics groups and hospitals staff in Muscat. In the first page of the survey, information about the study (such as aim of study and eligible participant) was given. Electronic consent was obtained from all participants before starting survey. The questionnaire consisted of three sections. The first section contained data regarding age, gender, education, employment, and tobacco use. The second part contained a 14-item questionnaire related to the MedDiet and third section collected information regarding anthropometric measurements. Participants were supported with photographs to identify food portion sizes. Participants were also provided with photographs to indicate how to measure WC and HC appropriately.

### 2.3. Adherence to Mediterranean Diet Questionnaire

A self-administrated questionnaire was distributed to the participants. The questionnaire was adapted from a validated 14-item questionnaire of Mediterranean diet adherence [30] and was used to measure adherence to the MedDiet among the study participants. This validated questionnaire was developed by The Prevención con Dieta Mediterránea (PREDIMED) study, which is the first trial to randomize high-risk individuals to follow either one of two Mediterranean diets or a control diet for primary cardiovascular disease prevention. This questionnaire was used in the baseline of the study to assess the adherence of the MedDiet. Furthermore, a full-length 137-item validated food frequency questionnaire (FFQ) was provided to PREDIMED study participants [34,35]. Given that, the difficulty of using a full length FFQ in our current study, we used the 14-item questionnaire which contained a scoring system. Only one question was eliminated from the questionnaire (question about wine intake), as alcohol beverages are prohibited in Islamic regions. The questionnaire was translated into Arabic. Each question was given a score of 0 or 1, score 1 denoting that the answer was in line with the MedDiet recommendations. Finally, a sum of scores from the 13 questions were obtained for all participants in order to define whether participant where low (0–5), medium (6–7), or high (8–13) adherence to MedDiet.

### 2.4. Anthropometric Measurement

Height was measured to the nearest centimeter using a calibrated measuring rod, when the subject was in a standing position without shoes. Weight was measured by using a portable electronic weight scale, while participants were wearing very light clothes, not wearing socks or shoes, and having an empty bladder. BMI was calculated as body weight in kilograms divided by height in meters squared (kg/m^2^). WC was measured at the midpoint between the lower border of the ribcage and the iliac crest using a non-elastic tape measure, while HC was measured at the widest part of the hips. Then, WHR calculated as follow (WHR = waist circumference ÷ hip circumference).

### 2.5. Statistical Analysis

Statistical analyses were performed using SPSS version 26.0 software. One-way analysis of variance (ANOVA) was used to analyze differences in baseline characteristics between participants from the three country. Chi square test was used to detect any differences in the level of adherence among study participants with specific characteristics. The association between adherence to MedDiet and BMI, WC, HC, and WHR were tested using liner regression. Estimated means were adjusted for cofounding factors age, sex, country of residence, education, smoking, and BMI. All reported probability tests *p* < 0.05 were considered significant. Values for continuous variables in the text and tables were mean ± SE; categorical variables were frequency and percentage.

## 3. Results

The participants main characteristics were showed in (Table 1), 75.7% of participants were women. There were significant differences in almost all of characteristics, except WC between the three Gulf countries (*p* < 0.0001). Saudi participants were younger in age (27.4 ± 9.6 years) and had a lower weight (64.8 ± 17.8 kg), BMI (24.3 ± 5.8 kg/m^2^), and HC (85.8 ± 18.3 cm) compared to Omani and Kuwaiti participants (Omani age 36.8 ± 8.2 years, weight 70.4 ± 16.0 kg, BMI 27.1 ± 5.9 kg/m^2^, and HC 95.0 ± 26.1 cm; Kuwaiti age 32.4 ± 11.2 years, weight 73.1 ± 17.0 kg, BMI 27.1 ± 5.5 kg/m^2^, and HC 101.3 ± 29.6 cm). The majority of participants were bachelor’s degree holders, non-smoker, and employed except in Saudis.

### 3.1. Pattern of Adherence to MedDiet among Participants from Gulf Countries

The mean score of the adherence to MedDiet among three gulf countries is showed in Table 2. The mean of the adherence to MedDiet score was 5.9 ± 2.03 for the total sample. Saudi participants had a significantly lower adherence to MedDiet score compared to Omani and Kuwaiti participants (*p* = 0.01). The majority of participants displayed a lower adherence to MedDiet with mean score 4.4 ± 1 in low group (44.4%), 6.4 ± 0.5 in medium group (33.1%), and 8.7 ± 0.8 in high group (22.4%). The adherence to the MedDiet in women was 6.04 ± 1.98, which was significantly higher than men (5.46 ± 2.11) (*p* = 0.0002). Participants with postgraduate degree (6.50 ± 2.02) showed more compliance to the MedDiet compared to bachelor degree holders (5.85 ± 2.02, *p* = 0.001) or individuals with lower education levels (5.65 ± 2, *p* = 0.00005).

The participants intake of the 13 food items is represented in Table 3 and is stratified by a MedDiet score as low (0–5), medium (6–7), or high (8–13). Around 85% in high and 59% in medium score groups reported the consumption olive oil; however, only 52% and 22% reached the recommended level of olive oil consumption (four teaspoons) in high and medium score levels, respectively. Most of participants failed to comply with recommendations of fruit and vegetable in low and medium score groups compared to the high score group. The consumption of red meat, butter, carbonated drinks, and poultry were in line with MedDiet recommendation in the majority of participant in all tertile groups; however, intake from legumes, fish, sweets, and nuts were not comply to optimal amount according to MedDiet recommendation.

### 3.2. Association between Adherence to MedDiet Score and Anthropometrics Measurment

An inverse association was observed between adherence to MedDiet and BMI (*p* = 0.0003) after adjusting for potential confounders (Table 4), where participants at the lower group (26.1 ± 6.7 kg/m^2^) had higher BMI compared to medium (25.5 ± 5.2 kg/m^2^) and high (25.0 ± 5.3 kg/m^2^) groups. Adherence to the MedDiet was significantly associated with a decreased risk of obesity (OR: −0.16; 95% CI: 0.61–1.18 and OR: −0.57; 95% CI: 0.56–0.38) when comparing medium and high tertiles, respectively, with the lowest tertile, after multivariable adjustment (*p* = 0.004). Stratified data based on gender showed that women with high adherence to MedDiet had significantly lower BMI (24.6 ± 5.2 kg/m^2^) compared to lower adherence group (*p* = 0.001). A protective effect was seen with a higher adherence to MedDiet on HC, suggesting that a greater adherence to the MedDiet was associated with a decreased HC (*p* = 0.04 in total participants, and *p* = 0.01 in women only). However, a higher adherence to the MedDiet (0.98 ± 0.21 cm) was positively associated with WHR level, in an adjusted model with women only, in comparison compared to women in the low (0.95 ± 0.20 cm) and medium (0.94 ± 0.23 cm) tertiles (*p* = 0.03). There was no evidence of an association between the adherence to MedDiet and higher WC.

## 4. Discussion

This is the first study to examine the adherence of the MedDiet in Gulf countries. This study used a scoring method based on validated questionnaire and examined the association of the adherence to MedDiet with several obesity indicators. Participants from Gulf countries in this study showed a low adherence to MedDeit and failed to comply with the recommendation of consuming olive oil, fruit and vegetables. Interestingly, among participants with the highest adherence to the MedDiet, there was a significant decrease in two obesity indicators, BMI, and HC. However, higher adherence to MedDiet was associated with increased WHR level, among women only. Therefore, we would have expected a lower incidence of obesity among participants with higher adherence to MedDiet.

Gulf countries have established food based dietary guidelines, which are available either in the Ministry of Health website or in the Food and Agriculture Organization website. These guidelines provide recommendations for an overall healthy dietary pattern for the general population based on food groups, such as 2–4 serving from fruit, 3–5 serving from vegetables, 2–3 serving from meat (red meat, chicken, and fish), as well for dairy products, grain, and oil, especially unsaturated fat and omega−3 from foods such as nuts, flaxseed, and fish. These recommendations take into account the availability of food and cultural acceptance within the countries [36,37,38]. These recommendations in some parts are in line with Mediterranean nutritional recommendations, which are considered an affordable and accessible dietary pattern [39]. Furthermore, some Mediterranean cuisine and dishes have been transformed and adopted by different regions around the world, i.e., Gulf countries have had migration of Eastern Mediterranean inhabitants from Syria, Palestine, and Lebanon. These dishes have been based on the MedDiet principles such as high intake fruit and vegetable, olive oil, grains, and legumes [40]. These reasons along with the beneficial health effects of MedDiet, may be encourage inhabitants of Gulf countries to follow this dietary pattern.

Several studies have been previously conducted in European countries to assess adherence to MedDiet, especially countries on Mediterranean coast. In a large trial (PREDIMED trial) conducted on 7447 participants from Spain (4282 women and 3165 men) the mean adherence score was 8.6 for total sample, which was relatively higher than score observed in our study [30]. Another large cohort from Spain (*n* = 13,380) assessed adherence to the MedDiet using a nine-scale index, 71% of participants had moderate adherence (score range from 3 to 6), 16% had low adherence (score below 2), 11% had high adherence (score range 7–9) [29]. Another cohort conducted on 32,119 Italian participants, where adherence to the MedDiet was assessed using an Italian index based on 11 items. Results have shown that 40% of participants had a low adherence (score below 3), 37% had moderate adherence (score range from 4 to 5), 22% had high adherence (score range 6–11) [27]. Findings from Lebanese participants, one of the most prominent Arab populations known by their Mediterranean cuisine, showed a low adherence to MedDiet with mean score of 4.2, based on scale of 10-items [41]. Dietary patterns among Gulf countries are characterized by high consumption of grain-based foods, mainly rice and wheat (which is used to produce bread, pastries, and other products), insufficient intake from fruit and vegetable, increasing intakes of animal products, with intake of red meat and poultry being consumed more than fish [37]. Additionally, the nutritional transition towards a more Western diets among Gulf countries citizen (high in animal derived-food products, added sugar, fat, salt and low plant food such as fruit and vegetables) [42], could explain the possible reasons for the low score seen in this study. These shifts in food consumption habits have been associated with an increase in urbanization, economic and technological development, and modernization in most countries of the region [42,43].

Our findings are consistent with a pervious study conducted in 265 participants from Saudi Arabia, which showed that 63% of study participants use olive oil, however, only 20% met the optimal amount according to Mediterranean recommendation. Furthermore, only 33% and 42% of participants consumed optimal amounts from fruit and vegetables, respectively [33]. Another cross-sectional study conducted on 200 Saudi adults assessed olive oil consumption from 24-h recall and the average olive oil consumption was 19 g/day for men and 17 g/day for women, which equates to 1.2 and 1.1 table spoon, respectively [44]. Previous studies on adults from Saudi and Kuwaiti population confirmed low consumption from fruit and vegetables (below five serving per day) [45,46], while the Omani population showed adequate intake from fruit but not from vegetables [15]. Among the Gulf region, olive tree cultivation is growing in Saudi Arabia. The north region of Saudi Arabia, mainly Aljouf, Tabouk, and Hail cities, are successful in olive oil production due to its climate and geographical factors. Over 13 million olive trees have been planted in these areas, increasing the accessibility of olive oil among locals [47]. Moreover, the food supply for cereal, fruit, meat, milk, and vegetables have scored highly among Gulf countries [43]; thus, food security is not considered an issue among these countries [48] and adaption of healthy dietary patterns such as the MedDiet will not impose added expense to the household.

Findings from the current study revealed that the highest adherence to the MedDiet was associated with the decrease of two obesity indicators, BMI and HC. In line with our findings, results from Italian cohort demonstrated that a higher adherence to the MedDiet was associated with a reduced risk of becoming overweight or obese, smaller five-year change in WC, and lower risk of abdominal obesity [27]. In a cross-sectional study conducted on 1048 Japanese adults, it was found that a higher adherence to the MedDiet reduced the risk of becoming overweight or obese. The study also demonstrated that an increase of two points in the MedDiet score decreased the risk of becoming overweight or obese by 0.76, this study was based on a Japanese-adapted MedDiet score containing 13-items [49]. However, adherence to the MedDiet was not associated with changes in WC in a large prospective cohort [50], which is in line with our findings. In addition, the results of this study showed that a higher adherence to the MedDiet were associated with an increased WHR level among women participants only. This might be due to the complexity of this tool as it involves two measurements (hip and waist); thus, there is more risk for measurement error [4].

One of mechanism that explain the relationship of MedDiet with obesity and weight status is its role in satiety. The MedDiet is high in fruit and vegetable, which are less dense in energy, high in fiber; thus, it enhances satiety [51,52,53]. Additionally, the high contents of polyphenols in MedDiet from extra-virgin olive oil, nuts, legume, whole grain, fruit, and vegetables, play several roles in weight management. For example, polyphenols activate β-oxidation, stimulate energy expenditure by inducing thermogenesis in brown adipose tissue, promoting adipocyte apoptosis and increasing lipolysis [54].

This study has some limitations. The study consists of a small sample size, hence creating a biased analysis and is not enough to represent the population. The cross-sectional study design can be another limitation for this study as it does not demonstrate causality unlike other prospective study designs. In contrast, the current study has also several strengths. Firstly, a validated 14-item questionnaire was used. Furthermore, using several obesity indicators to assess obesity reflect accurate results about the prevalence of obesity among the study sample rather than using one measurement such as BMI. Moreover, this is the first study to compare the adherence to the MedDiet among adults from three Gulf countries (Saudi Arabia, Oman, and Kuwait) and to examine its association with increased risk of obesity using several obesity indicators (BMI, WC, HC, and WHR).

## 5. Conclusions

The results of this study indicate a low adherence to the MedDiet among participants from three gulf countries. A significant negative association was found between higher adherence to MedDiet and two obesity indicators, BMI, and HC. This indicates that that the MedDiet approach could be useful in reducing the risk of obesity in gulf countries. Nutrition and dietetic societies among Gulf countries should implement programs to increase public awareness of the health benefits of following healthy dietary patterns. Furthermore, studies utilizing a larger sample size with more biochemical analysis to address the effect of adherence to MedDiet or other healthy dietary patterns on diabetes and cardiovascular disease among Gulf populations are needed.

## Figures and Tables

**Table 1:** Main characterization of study sample.

Characteristics	Total (*n* = 961)	Saudi Participants (*n* = 496)	Omani Participants (*n* = 335)	Kuwaiti Participants (*n* = 130)	*p* Value
Age (years)	31.3 ± 10.3	27.4 ± 9.6	36.8 ± 8.2	32.4 ± 11.2	<0.0001
**Gender**					<0.0001
Men	234 (24.3)	163 (32.9)	51 (15.2)	20 (15.4)	
Women	727 (75.7)	333 (67.1)	284 (84.8)	110 (84.6)	
Weight (kg)	67.8 ± 17.3	64.8 ± 17.8	70.4 ± 16.0	73.1 ± 17.0	<0.0001
Body mass index (kg/m^2^)	25.6 ± 6.0	24.3 ± 5.8	27.1 ± 5.9	27.1 ± 5.5	<0.0001
Waist circumference (cm)	87.5 ± 20.4	86.8 ± 18.4	88.7 ± 22.2	87.2 ± 24.0	0.48
Hip circumference (cm)	90.8 ± 23.4	85.8 ± 18.3	95.0 ± 26.1	101.3 ± 29.6	<0.0001
Waist to hip ratio	0.9 ± 0.2	1.0 ± 0.2	0.9 ± 0.2	0.8 ± 0.2	<0.0001
**Education**					<0.0001
High school or lower	258 (26.8)	168 (33.9)	37 (11.0)	53 (40.8)	
Undergraduate	556 (57.9)	312 (62.9)	187 (55.8)	57 (43.8)	
Postgraduate	147 (15.3)	16 (3.2)	111 (33.1)	20 (15.4)	
**Employment**					<0.0001
Students	311 (32.4)	256 (51.6)	17 (5.1)	38 (29.2)	
Employee	447 (46.5)	159 (32.1)	219 (65.4)	69 (53.1)	
Unemployed	176 (18.3)	75 (15.1)	82 (24.5)	19 (14.6)	
Retired	27 (2.8)	6 (1.2)	17 (5.1)	4 (3.1)	
**Smoking status**					<0.0001
Smoker	131 (13.6)	92 (18.5)	9 (2.7)	30 (23.1)	
Non-smoker	830 (86.4)	404 (81.5)	326 (97.3)	100 (76.9)	

Continuous data are represented as mean ± SD and categorical variables represented as *n* (%). Statistical differences in characteristics between countries was tested by ANOVA for continues variable and chi square for categorical variable.

**Table 2:** Adherence to Mediterranean diet mean score among three gulf countries.

Total	Saudi	Omani	Kuwaiti	*p* Value
5.9 ± 2.03	5.7 ± 2.1	6.1 ± 1.9	6.1 ± 1.9	0.018

**Table 3:** Frequencies of 14-item questionnaire response according to level of adherence to the Mediterranean diet (MedDiet) in total sample size.

Questions	Low	Medium	High	*p* Value
Olive oil consumption				<0.0001
Yes	89 (20.8)	188 (59.1)	184 (85.2)	
No	338 (79.2)	130 (40.9)	32 (14.8)	
Amount of olive oil				<0.0001
<4 tps	404 (94.6)	248 (78.0)	103 (47.7)	
≥4 tps	23 (5.4)	70 (22.0)	113 (52.3)	
Vegetables				<0.0001
<2 servings	382 (89.5)	236 (74.2)	72 (33.3)	
≥2 servings	45 (10.5)	82 (25.8)	144 (66.7)	
Fruit				<0.0001
<3 servings	398 (93.2)	269 (84.6)	115 (53.2)	
≥3 servings	29 (6.8)	49 (15.4)	101 (46.8)	
Red meat				<0.0001
<1 serving	232 (54.3)	244 (76.7)	160 (74.1)	
>1 serving	195 (45.7)	74 (23.3)	56 (25.9)	
Butter				<0.0001
<1 serving	271 (63.5)	270 (84.9)	192 (88.9)	
>1 serving	156 (36.5)	48 (15.1)	24 (11.1)	
Carbonated drinks				<0.0001
<1 cup	275 (64.4)	267 (84.0)	197 (91.2)	
>1 cup	152 (35.6)	51 (16.0)	19 (8.8)	
Legumes				<0.0001
<3 servings	345 (80.8)	205 (64.5)	121 (56.0)	
≥3 servings	82 (19.2)	113 (35.5)	95 (44.0)	
Fish				<0.0001
<3 servings	371 (86.9)	242 (76.1)	112 (51.9)	
≥3 servings	56 (13.1)	76 (23.9)	104 (48.1)	
Cakes and sweets				<0.0001
<3 servings	145 (34.0)	173 (54.4)	157 (72.7)	
≥3 servings	282 (66.0)	145 (45.6)	59 (27.3)	
Nuts				<0.0001
<3 servings	366 (85.7)	228 (71.7)	107 (49.5)	
≥3 servings	61 (14.3)	90 (28.3)	109 (50.5)	
Poultry				<0.0001
Yes	245 (57.5)	264 (83.0)	187 (86.6)	
No	182 (42.6)	54 (17.0)	29 (13.4)	
Tomato sauce				<0.0001
<2 servings	243 (56.9)	154 (48.4)	75 (34.7)	
≥2 servings	184 (43.1)	164 (51.6)	141 (65.3)	

Statistical differences were tested by chi square.

**Table 4:** Association between adherence to MedDiet score and obesity risk factors.

	Low Score	Medium Score	High Score	Adjusted *p* Value
BMI				
Total	26.1 ± 6.7	25.5 ± 5.2	25.0 ± 5.3	0.0003
Men	26.5 ± 4.9	26.7 ± 5.2	26.8 ± 5.9	0.47
Women	25.9 ± 7.4	25.2 ± 5.2	24.6 ± 5.2	0.001
WC				
Total (688)	86.9 ± 23.2	87.8 ± 20.2	86.0 ± 19.2	0.53
Men	93.8 ± 20.3	100.3 ± 16.7	96.3 ± 18.7	0.64
Women	84.1 ± 23.8	84.4 ± 19.8	83.3 ± 18.4	0.77
HC				
Total	90.1 ± 25.1	92.7 ± 23.4	88.5 ± 20.8	0.04
Men	91.1 ± 15.8	98.6 ± 19.6	94.9 ± 16.4	0.11
Women	89.6 ± 28.2	91.1 ± 24.1	86.8 ± 21.6	0.01
WHR				
Total	0.97 ± 0.19	0.96 ± 0.22	0.98 ± 0.20	0.10
Men	1.02 ± 0.15	1.03 ± 0.16	1.01 ± 0.15	0.60
Women	0.95 ± 0.20	0.94 ± 0.23	0.98 ± 0.21	0.03

Adjusted *p* value was calculated by linear regression model adjusted for age, country of residence, sex, education level, BMI, and smoking status.

## References

[1] Finkelstein E.A., Khavjou O.A., Thompson H., Trogdon J.G., Pan L., Sherry B., Dietz W. (2012). Obesity and severe obesity forecasts through 2030. Am. J. Prev. Med..

[2] WHO Expert Consultation (2004). Appropriate body-mass index for Asian populations and its implications for policy and intervention strategies. Lancet.

[3] Ramachandran A., Snehalatha C. (2010). Rising burden of obesity in Asia. J. Obes.

[4] Al-Sharbatti S., Shaikh R., Mathew E., Sreedharan J., Muttappallymyalil J., Basha S. (2011). The Use of Obesity Indicators for the Prediction of Hypertension Risk among Youth in the United Arab Emirates. Iran. J. Public Health.

[5] Balhareth A., Meertens R., Kremers S., Sleddens E. (2019). Overweight and obesity among adults in the Gulf States: A systematic literature review of correlates of weight, weight-related behaviours, and interventions. Obes. Rev..

[6] D’Innocenzo S., Biagi C., Lanari M. (2019). Obesity and the Mediterranean Diet: A Review of Evidence of the Role and Sustainability of the Mediterranean Diet. Nutrients.

[7] Al-Qahtani M.H. (2016). Dietary Habits of Saudi Medical Students at University of Dammam. Int. J. Health Sci..

[8] Syed N.K., Syed M.H., Meraya A.M., Albarraq A.A., Al-Kasim M.A., Alqahtani S., Makeen H.A., Yasmeen A., Banji O.J.F., Elnaem M.H. (2020). The association of dietary behaviors and practices with overweight and obesity parameters among Saudi university students. PLoS ONE.

[9] Benajiba N. (2016). Fast food intake among Saudi population: Alarming fact. Am. J. Food Nutr..

[10] Amin T.T., Al-Sultan A.I., Ali A. (2008). Overweight and Obesity and their Association with Dietary Habits, and Sociodemographic Characteristics Among Male Primary School Children in Al-Hassa, Saudi Arabia. Indian J. Community Med..

[11] Tamimi J. (2016). Consumption of Whole Grains by a Sample of Saudi Adults. Int. J. Food Sci. Nutr..

[12] Moradi-Lakeh M., El Bcheraoui C., Afshin A., Daoud F., AlMazroa M.A., Al Saeedi M., Basulaiman M., Memish Z.A., Al Rabeeah A.A., Mokdad A.H. (2017). Diet in Saudi Arabia: Findings from a nationally representative survey. Public Health Nutr..

[13] Al-Lahou B., Ausman L.M., Peñalvo J.L., Huggins G.S., Al-Hooti S., Al-Zenki S., Zhang F.F. (2020). Dietary Patterns Associated with the Prevalence of Cardiovascular Disease Risk Factors in Kuwaiti Adults. J. Acad. Nutr. Diet..

[14] Shaban L., Alkazemi D. (2019). Trends in Fast-food Consumption among Kuwaiti Youth. Int. J. Prev Med..

[15] Al Riyami A., Al Hadabi S., Abd El Aty M.A., Al Kharusi H., Morsi M., Jaju S. (2010). Nutrition knowledge, beliefs and dietary habits among elderly people in Nizwa, Oman: Implications for policy. East. Mediterr. Health J..

[16] Anwar S., Al Adawi A.H.A., Al-Zubaidi N., Al Kharousi M.N.R., Al-Jahwari J.A.S., Samad R.A. (2018). Food eating habits of adolescents in Oman. Ann. Trop Med. Public Health.

[17] Mehdi I., Al Bahrani B. (2020). Fast Food Chains and Obesity in Oman-Commentary Article. J. Cancer Epidemiol. Prev..

[18] Majeed F. (2015). Association of BMI with diet and physical activity of female medical students at the University of Dammam, Saudi Arabia. J. Taibah Univ. Med. Sci.

[19] Musaiger A. (2011). Overweight and Obesity in Eastern Mediterranean Region: Prevalence and Possible Causes. J. Obes.

[20] Lassale C., Fezeu L., Andreeva V.A., Hercberg S., Kengne A.P., Czernichow S., Kesse-Guyot E. (2012). Association between dietary scores and 13-year weight change and obesity risk in a French prospective cohort. Int. J. Obes..

[21] Wirt A., Collins C.E. (2009). Diet quality--what is it and does it matter?. Public Health Nutr..

[22] Hu F.B. (2002). Dietary pattern analysis: A new direction in nutritional epidemiology. Curr. Opin. Lipidol..

[23] Bach-Faig A., Berry E.M., Lairon D., Reguant J., Trichopoulou A., Dernini S., Medina F.X., Battino M., Belahsen R., Miranda G. (2011). Mediterranean diet pyramid today. Science and cultural updates. Public Health Nutr..

[24] Sofi F., Macchi C., Abbate R., Gensini G.F., Casini A. (2013). Mediterranean diet and health. BioFactors.

[25] Dinu M., Pagliai G., Casini A., Sofi F. (2018). Mediterranean diet and multiple health outcomes: An umbrella review of meta-analyses of observational studies and randomised trials. Eur. J. Clin. Nutr..

[26] Huo R., Du T., Xu Y., Xu W., Chen X., Sun K., Yu X. (2015). Effects of Mediterranean-style diet on glycemic control, weight loss and cardiovascular risk factors among type 2 diabetes individuals: A meta-analysis. Eur. J. Clin. Nutr..

[27] Agnoli C., Sieri S., Ricceri F., Giraudo M.T., Masala G., Assedi M., Panico S., Mattiello A., Tumino R., Giurdanella M.C. (2018). Adherence to a Mediterranean diet and long-term changes in weight and waist circumference in the EPIC-Italy cohort. Nutr. Diabetes.

[28] Beunza J.J., Toledo E., Hu F.B., Bes-Rastrollo M., Serrano-Martínez M., Sánchez-Villegas A., Martínez J.A., Martínez-González M.A. (2010). Adherence to the Mediterranean diet, long-term weight change, and incident overweight or obesity: The Seguimiento Universidad de Navarra (SUN) cohort. Am. J. Clin. Nutr..

[29] Martínez-González M.A., de la Fuente-Arrillaga C., Nunez-Cordoba J.M., Basterra-Gortari F.J., Beunza J.J., Vazquez Z., Benito S., Tortosa A., Bes-Rastrollo M. (2008). Adherence to Mediterranean diet and risk of developing diabetes: Prospective cohort study. BMJ.

[30] Martínez-González M.A., García-Arellano A., Toledo E., Salas-Salvadó J., Buil-Cosiales P., Corella D., Covas M.I., Schröder H., Arós F., Gómez-Gracia E. (2012). A 14-item Mediterranean diet assessment tool and obesity indexes among high-risk subjects: The PREDIMED trial. PLoS ONE.

[31] Filippatos T.D., Panagiotakos D.B., Georgousopoulou E.N., Pitaraki E., Kouli G.M., Chrysohoou C., Tousoulis D., Stefanadis C., Pitsavos C., ATTICA Study Group (2016). Mediterranean Diet and 10-year (2002–2012) Incidence of Diabetes and Cardiovascular Disease in Participants with Prediabetes: The ATTICA study. Rev. Diabet Stud..

[32] Khalili-Moghadam S., Mirmiran P., Bahadoran Z., Azizi F. (2019). The Mediterranean diet and risk of type 2 diabetes in Iranian population. Eur. J. Clin. Nutr..

[33] Aljabri M., Al-Raddadi R., Bahijri S.M., Al Ahmadi J., Ajabnoor G., Jambi H.A. (2019). Factors associated with adherence to Mediterranean diet among Saudi non-diabetic patients attending primary health care centers: A cross-sectional study. J. Taibah Univ. Med. Sci..

[34] Martínez-González M., Buil-Cosiales P., Corella D., Bulló M., Fitó M., Vioque J., Romaguera D., Martínez J.A., Wärnberg J., López-Miranda J. (2012). Cohort profile: Design and methods of the PREDIMED study. Int. J. Epidemiol..

[35] Fernández-Ballart J.D., Piñol J.L., Zazpe I., Corella D., Carrasco P., Toledo E., Perez-Bauer M., Martínez-González M.A., Salas-Salvadó J., Martín-Moreno J.M. (2010). Relative validity of a semi-quantitative food-frequency questionnaire in an elderly Mediterranean population of Spain. Br. J. Nutr..

[36] Montagnese C., Santarpia L., Iavarone F., Strangio F., Sangiovanni B., Buonifacio M., Caldara A.R., Silvestri E., Contaldo F., Pasanisi F. (2019). Food-Based Dietary Guidelines around the World: Eastern Mediterranean and Middle Eastern Countries. Nutrients.

[37] Musaiger A.O., Takruri H.R., Hassan A.S., Abu-Tarboush H. (2012). Food-based dietary guidelines for the arab gulf countries. J. Nutr. Metab..

[38] Coats L., Bernstein J., Dodge E., Bechard L., Aboul-Enein B.H. (2019). Food-based dietary guidelines of Arabic-speaking countries: A culturally congruent profile. Public Health Nutr..

[39] Saulle R., Semyonov L., La Torre G. (2013). Cost and cost-effectiveness of the Mediterranean diet: Results of a systematic review. Nutrients.

[40] Renna M., Rinaldi V.A., Gonnella M. (2015). The Mediterranean Diet between traditional foods and human health: The culinary example of Puglia (Southern Italy). Int. J. Gastron. Food Sci..

[41] Farhat A., Jaalouk D., Francis S. (2016). Adherence to the Mediterranean diet in a Lebanese sample. Nutr. Food Sci..

[42] Popkin B.M., Adair L.S., Ng S.W. (2012). Global nutrition transition and the pandemic of obesity in developing countries. Nutr. Rev..

[43] Ahmed A., Elbushra A., Salih O. (2019). Food Consumption Patterns and Trends in the Gulf Cooperation Council. Pak. J. Nutr.

[44] AlKhattaf N.F., Alraddadi A.M., Aljarbou M.A., Arnauti M.A., Alfaleh A.M., Hammouda S.A. (2020). Determining the correlation between olive oil consumption, BMI, and waist circumference in the adult Saudi population. J. Taibah Univ. Med. Sci..

[45] Al-Otaibi H.H. (2013). The pattern of fruit and vegetable consumption among Saudi university students. Glob. J. Health Sci..

[46] Zaghloul S., Waslien C., Al Somaie M., Prakash P. (2012). Low adherence of Kuwaiti adults to fruit and vegetable dietary guidelines. East. Mediterr. Health J..

[47] Sabouni I. (2018). Physicochemical characterization of olive oil from Aljouf area of Saudi Arabia. Int. J. Chemtech. Res..

[48] Ben Hassen T., El Bilali H. (2019). Food Security in the Gulf Cooperation Council Countries: Challenges and Prospects. J. Food Secur..

[49] Kanauchi M., Kanauchi K. (2016). Development of a Mediterranean diet score adapted to Japan and its relation to obesity risk. Food Nutr. Res..

[50] Li Y., Roswall N., Ström P., Sandin S., Adami H.O., Weiderpass E. (2015). Mediterranean and Nordic diet scores and long-term changes in body weight and waist circumference: Results from a large cohort study. Br. J. Nutr.

[51] Drewnowski A., Almiron-Roig E., Marmonier C., Lluch A. (2004). Dietary energy density and body weight: Is there a relationship?. Nutr. Rev..

[52] Rolls B., Ello-Martin J., Tohill B. (2004). What Can Intervention Studies Tell Us about the Relationship between Fruit and Vegetable Consumption and Weight Management?. Nutr. Rev..

[53] Yuan S., Yu H.J., Liu M.W., Huang Y., Yang X.H., Tang B.W., Song Y., Cao Z.K., Wu H.J., He Q.Q. (2018). The association of fruit and vegetable consumption with changes in weight and body mass index in Chinese adults: A cohort study. Public Health.

[54] Castro-Barquero S., Lamuela-Raventós R.M., Doménech M., Estruch R. (2018). Relationship between Mediterranean Dietary Polyphenol Intake and Obesity. Nutrients.

